# Perspective: Peer Evaluation of Recommendations for CONSORT Guidelines for Randomized Controlled Trials in Nutrition^☆^

**DOI:** 10.1016/j.advnut.2023.100154

**Published:** 2023-11-21

**Authors:** Connie Weaver, Sanne Ahles, Karen J. Murphy, Sangeetha Shyam, Janet Cade, Jogchum Plat, Lukas Schwingshackl, Helen M. Roche, Carl Lachat, Anne-Marie Minihane, Jessica Rigutto-Farebrother, Federation of Nutritional Sciences (FENS) Working Groups on Improving Standards in the Science of Nutrition

**Affiliations:** 1 School of Exercise and Nutritional Sciences, San Diego State University, San Diego, CA, United States; 2 Department of Nutrition and Movement Sciences, School of Nutrition and Translational Research in Metabolism (NUTRIM), Maastricht University, Maastricht, The Netherlands; 3 BioActor BV, Maastricht, The Netherlands; 4 Clinical and Health Sciences and Alliance for Research in Exercise, Nutrition and Activity University of South Australia, Adelaide, South Australia, Australia; 5 Centre for Translational Research, Institute for Research, Development, and Innovation (IRDI), International Medical University, Kuala Lumpur, Malaysia; 6 Universitat Rovira i Virgili, Department of Biochemistry and Biotechnology, Human Nutrition Unit, Biomedical Research Network Center for Physiopathology of Obesity and Nutrition (CIBEROBN), Carlos III Health Institute (ISCIII), Reus, Spain; 7 Nutritional Epidemiology Group, School of Food Science and Nutrition, University of Leeds, Leeds, United Kingdom; 8 Institute for Evidence in Medicine, Medical Center - University of Freiburg, Faculty of Medicine, University of Freiburg, Freiburg, Germany; 9 Nutrigenomics Research Group, UCD Conway Institute, School of Public Health, Physiotherapy and Sports Science, University College Dublin, Dublin, Ireland; 10 Institute for Global Food Security, The Queen’s University of Belfast, Belfast, Northern Ireland, United Kingdom; 11 Department of Food Technology, Safety and Health, Ghent University, Ghent, Belgium; 12 Nutrition and Preventive Medicine, Norwich Medical School, University of East Anglia, Norwich, United Kingdom; 13 Norwich Institute of Healthy Ageing, University of East Anglia, Norwich, United Kingdom; 14 Laboratory of Nutrition and Metabolic Epigenetics, Institute for Food, Nutrition and Health, ETH Zürich, Zürich, Switzerland; 15 Global Center for the Development of the Whole Child, University of Notre Dame, Notre Dame, IN, United States

**Keywords:** guidelines, CONSORT, nutrition trials, dietary interventions, reporting, expert opinion

## Abstract

Creating effective dietary guidance requires a rigorous evidence base that is predominantly developed from robust clinical trials or large-scale cohort studies, with the quality of the data available depending on the completeness and accuracy of their reporting. An international group of academics from 14 institutions in 12 different countries and on 5 continents, working on behalf of the Federation of European Nutrition Societies within its “Improving Standards in the Science of Nutrition” initiative, reviewed the Consolidated Standards of Reporting Trials (CONSORT) statement checklist as it pertains to nutrition trials. This perspective piece documents the procedure followed to gain input and consensus on the checklist previously published by this group, including its presentation and interrogation at the International Union of Nutritional Sciences International Congress of Nutrition 2022 (IUNS-ICN 22), inputs from a survey of journal editors, and its piloting on 8 nutrition trials of diverse designs. Overall, the initiative has been met with considerable enthusiasm. At IUNS-ICN 22, refinements to our proposal were elicited through a World Café method discussion with participating nutrition scientists. The contributing journal editors provided valuable insights, and the discussion led to the development of a potential tool specific to assess adherence to the proposed nutrition extension checklist. The piloting of the proposed checklist provided evidence from real-life studies that reporting of nutrition trials can be improved. This initiative aims to stimulate further discussion and development of a CONSORT-nutrition-specific extension.

**Statement of significance**

CONSORT provides general guidance on reporting the accuracy and completeness of randomized controlled trials. Many discipline-specific extensions to CONSORT guidelines exist, but none for nutrition trials. Following on from our earlier work, this article describes refinements to the draft nutrition extension, with significant input from several stakeholders.

## Introduction

Developing effective dietary guidance requires a rigorous evidence base. Several recent reports describe efforts aimed at enhancing the rigor of nutrition research [1,2]. With the aim of improved reporting of human nutrition trials and within its “Improving Standards in the Science of Nutrition” initiative, the Federation of European Nutrition Societies (FENS) called for the development of recommendations for a nutrition extension to the CONSORT statement [[3], [4], [5]].

FENS is a coalition of 26 nutrition societies across Europe to promote the advancement of nutrition science, research, and development through international cooperation. Working groups were formed to address priority areas resulting from a 2019 workshop in Dublin. To provide a more robust evidence base, an international working group was formed of representatives from 14 institutions in 12 different countries and on 5 continents to review the CONSORT statement. For an initial focus, we chose the checklist out of the CONSORT resources (that also include the text and flowchart), specifically as it applies to all types of nutrition intervention trials providing single foods or supplements, dietary advice, or behavioral studies as applied to primary outcomes and secondary analyses. The draft recommendations for a nutrition-specific extension to the 25-item CONSORT checklist have been published [6]. Here, we report an evaluation process of those recommendations, which has led to a proposal for their refinement, as a first step toward a larger and more formal decision and possible effort to develop new guidelines. We conducted this evaluation in 3 ways. First, we sought peer feedback from the nutrition community at the International Union of Nutritional Sciences International Congress of Nutrition 2022 (IUNS-ICN 22) through a dedicated symposium [7]. This step is important because guidance for preparing new guidelines includes an assessment of interest from stakeholders [8] and engagement and involvement by prospective users from relative research communities from the outset.

We also presented our efforts through an oral abstract presentation at the conference, to inform the nutrition research community, and generate interest and engagement. Then, we canvassed opinions from global nutrition journal editors in an online meeting. Finally, we tested our recommendations on 8 trials of different designs, which were given a score based on how the parameters identified by the draft recommendations were reported.

**FIGURE 1:**
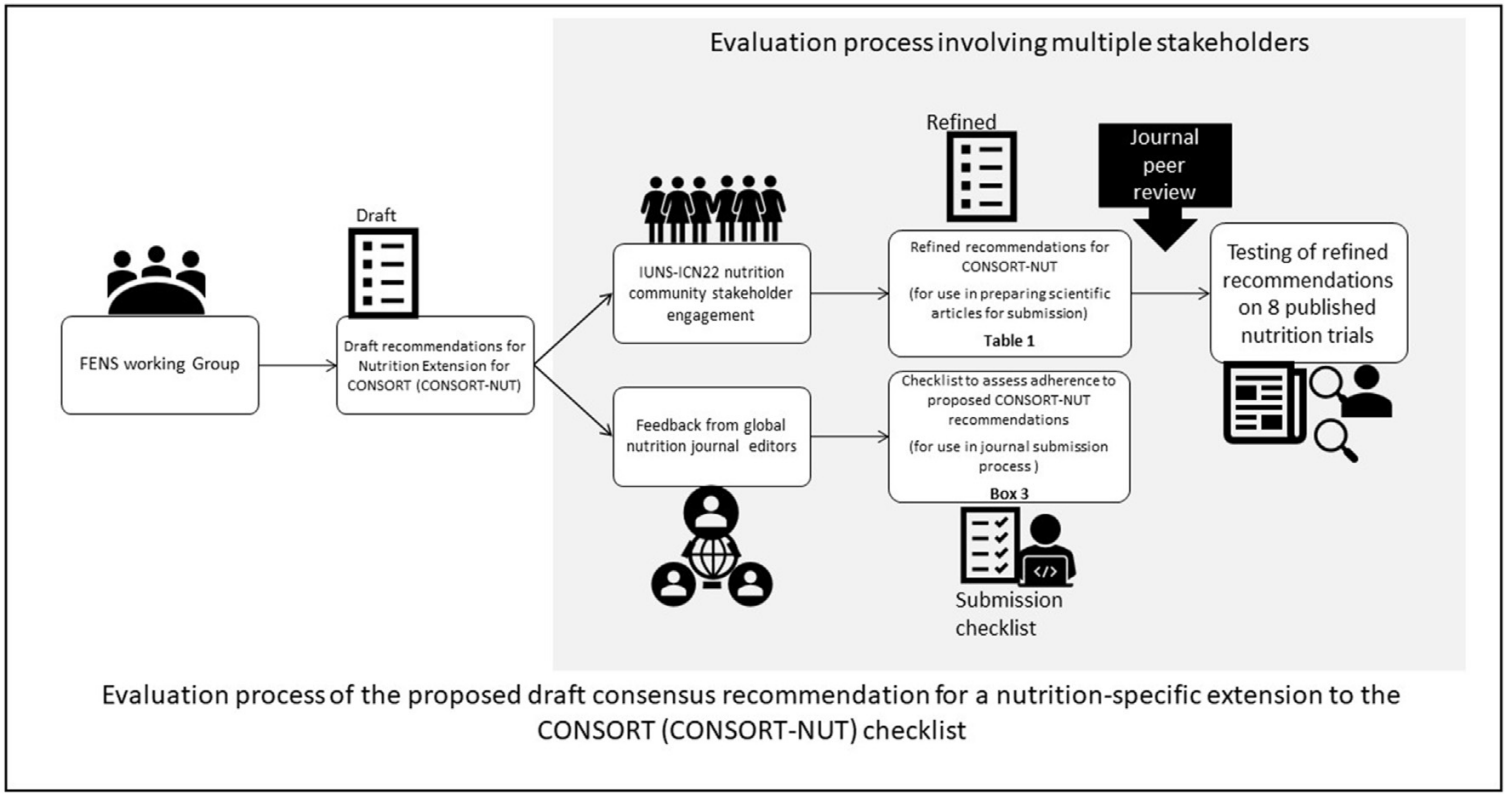
Overview of the evaluation process to date for the proposed draft consensus recommendations for a nutrition extension to CONSORT. IUNS-ICN 22, International Union of Nutritional Sciences International Congress of Nutrition 2022.

**FIGURE 2:**
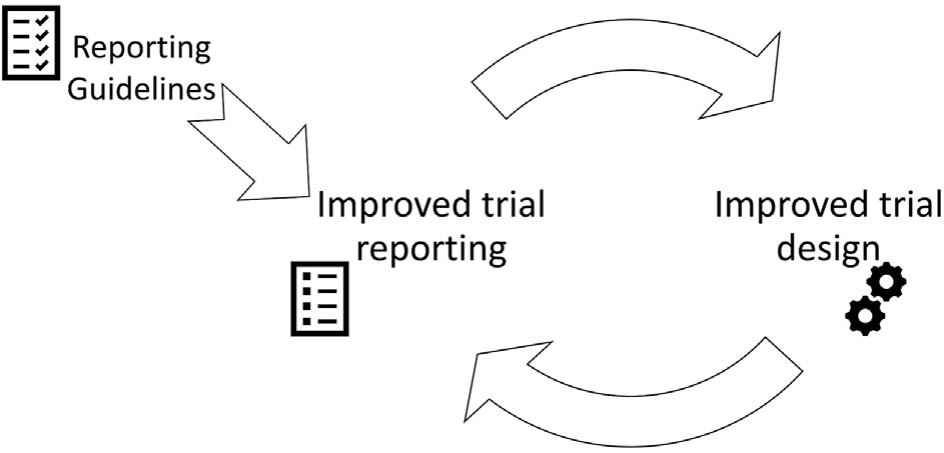
Rigorous reporting of trials feeds back to creating more rigorous study designs.

We acknowledge that reporting guidelines are a comprehensive set of recommendations on what to include in an article and that they also include graphical elements, such as a flowchart. For this initiative to date, we have focused on the content and use of the CONSORT checklist as a summary of the guidelines that are most used by authors and editors. The CONSORT checklist with the draft nutrition-specific recommendations as developed in our first manuscript [6] is shown in Table 1. In italics and bold are modifications resulting from the review of these recommendations by nutrition scientists (as described later in the section Input from a Preconference World Café Workshop at the International Congress of Nutrition 2022) and nutrition journal editors (as described in Input from nutrition journal editors), and the checklist has been further refined by reviewers of this manuscript. Our approach is summarized in Figure 1. It is also important to note that rigorous reporting of trials feeds back to more rigorous study designs (Figure 2). Currently, there are no standardized recommendations for rigorous study designs with systematic assessment as CONSORT provides for reporting of trials. Readers hence depend on the quality control at the reporting stage to feed back to investigators as they design future trials.

**TABLE 1:** Draft recommendations for nutrition-specific CONSORT reporting

Section/topic	Item no.			CONSORT checklist item		Recommendations for nutrition trials	

**Title and abstract**							
	1a			Identification as a randomized trial in the title		• Where possible, the type of dietary comparator should be described in the title, specifically “RCT” for trials with a control group, “trial” where 2 intervention groups are used and “placebo-controlled trial” where a placebo is used as comparator. • **One editor said distinguishing between an RCT and trial is not critical.**	
	1b			Structured summary of trial design, methods, results, and conclusions (for specific guidance see CONSORT for abstracts)		• Include details of the food bioactives, food/food group, dietary pattern, or eating behavior intervention *and comparator* • Clearly state if nutritional status, dietary intake, or eating behavior is the primary outcome • Specified the trial design, eg, cluster, cross-over, parallel, non-inferiority • *Include treatment effects* • State if the manuscript reports a secondary RCT analysis	
**Introduction**							
Background and objectives	2a			Scientific background and explanation of rationale		• State the biological plausibility of the nutrition intervention and**/or** behavioral, physiological, or molecular mechanism underpinning the intervention impact on the primary outcome measures • Provide contextualization *where relevant* to current dietary recommendations or food intake in the population of interest. Justify the population chosen giving details. Ensure PICO criteria are identifiable.	
	2b			Specific objectives*, aims, and* hypotheses			
**Methods**							
Trial design		3a			Description of trial design (such as parallel, factorial) including allocation ratio		• The trial design should align with the scientific question being addressed • Duration of the trial should be appropriate for the primary and key secondary *[nutrition sensitive]* outcomes • Potential **confounders** should be **described** including baseline nutritional status (especially for the nutrient, bioactive, diet being tested to determine if participants are already adequate) and factors that could influence nutrition trial outcomes (habitual diet, *socioeconomic status*, season, physical activity, knowledge of participants and interventionists, especially for education interventions), carry-over effects in cross-over trials
		3b			Important changes to methods after trial commencement (such as eligibility criteria), with reasons		
Participants		4a			Eligibility criteria for participants		• Target populations- clinical*, at-risk, and* healthy population, specify particular dietary, physiological, or nutritional characteristics targeted. List eligibility criteria related to baseline nutritional status (anthropometric, biochemical, clinical, diet, food allergies)
		4b			Settings and locations where the data were collected		
Interventions		5			The interventions for each group with sufficient details to allow replication, including how and when they were actually administered		• Dietary comparators should be well described, including details if isocaloric or not, as applicable. • Details of the diet-related intervention should be given. If given, how was it *prepared {form, matrix, co-ingested nutrients and constituents, food type, presentation (tablet, drink, food)}*, stored, checked for bioactive constituent(s), evaluated for storage stability, and biological exposure monitored? For behavioral interventions, describe the protocol that includes how it was developed and administered and by whom and when. A description of assessment of background diets is needed as relevant. • *Include acceptability and tolerance of intervention*
Outcomes		6a			Completely defined pre-specified primary and secondary outcome measures, including how and when they were assessed		• *Measure anticipated confounders*
		6b			Any changes to trial outcomes after the trial commenced, with reasons		
Sample size		7a			How sample size was determined		
		7b			When applicable, explanation of any interim analyses and stopping guidelines		
Randomization:							
• Sequence generation		8a			Method used to generate the random allocation sequence		• Report when randomization is based on nutrient intake or status
		8b			Type of randomization; details of any restriction (such as blocking and block size)		
• Allocation concealment		9			Mechanism used to implement the random allocation sequence (such as sequentially numbered containers), describing any steps taken to conceal the sequence until interventions were assigned		
• Implementation		10			Who generated the random allocation sequence, who enrolled participants, and who assigned participants to interventions		
Blinding		11a			If done, who was blinded after assignment to interventions (for example, participants, care providers, those assessing outcomes) and how		• Describe any limits to blinding and who was blinded (participants, staff who delivered the intervention, analytical staff), as well as details of concealed allocation
		11b			If relevant, a description of the similarity of interventions		
Statistical methods		12a			Statistical methods used to compare groups for primary and secondary outcomes		• An a priori statistical analysis plan that aligns with the study design should be described, and primary analysis should be based on intention-to-treat *[participants did not agree]*, with per-protocol analysis described in addition where relevant. • Comparisons between intention-to-treat and per-protocol analyses should be considered. Additionally, per-protocol compliance cutoffs should be reported, including possible exclusion criteria for misreporting • *Must adjust for stratification variables*
		12b			Methods for additional analyses, such as subgroup analyses and adjusted analyses		• Identify and justify data analysis choice (eg, statistical method used to combine dietary or nutritional data, energy adjustments, intake modeling, use of weighting factors). Define stratifications and adjustments • *Post-SAP analysis should be clearly identified as exploratory*
**Results**							
Participant flow (a diagram is strongly recommended)	13a			For each group, the numbers of participants who were randomly assigned, received intended treatment and were analyzed for the primary outcome			
	13b			For each group, losses and exclusions after randomization, together with reasons			
Recruitment	14a			Dates defining the periods of recruitment and follow-up			
	14b			Why the trial ended or was stopped			
Baseline data	15			A table showing baseline demographic and clinical characteristics for each group			
Numbers analyzed	16			For each group, the number of participants (denominator) included in each analysis and whether the analysis was by originally assigned groups			
Outcomes and estimation	17a			For each primary and secondary outcome, results for each group, and the estimated effect size and its precision (such as 95% confidence interval)			
	17b			For binary outcomes, presentation of both absolute and relative effect sizes is recommended			
Ancillary analyses	18			Results of any other analyses performed, including subgroup analyses and adjusted analyses, distinguishing pre-specified from exploratory			
Harms	19			All important harms or unintended effects in each group (for specific guidance see CONSORT for harms)			
Discussion							
Limitations	20			Trial limitations, addressing sources of potential bias, imprecision, and, if relevant, multiplicity of analyses			
Generalizability	21			Generalizability (external validity, applicability) of the trial findings			• Discuss generalizability with consideration to background diet and any variation in other populations, ensuring a differentiation between efficacy and effectiveness.
Interpretation	22			Interpretation consistent with results, balancing benefits and harms, and considering other relevant evidence			• State the main findings of the article, using intention-to-treat principles, with per-protocol interpretations given in addition, depending on the objective of the study. Provide a clear differentiation for these findings from ancillary analyses. • Discuss the choice of comparator, including whether isocaloric exchange was used or not, and any bias introduced. • Discuss any assessment of dietary adherence. • Discuss any relevant aspects of the active constituent of the intervention as revealed by the trial. • Describe any potentially false discoveries due to any adjustments used in statistical analyses. • Distinguish clearly between statistical and clinically relevant findings, with a detailed interpretation of how the findings affect clinical practice, dietary guidance, or public health recommendations, as relevant.
Other information							
Registration		23	Registration number and name of trial registry				
Protocol		24	Where the full trial protocol can be accessed, if available				
Funding		25	Sources of funding and other support (such as supply of drugs), role of funders				

*Italics: nutrition scientists at IUNS*; **Bold: journal editors.**

RCT, randomized controlled trial.

## Input from a Preconference World Café Workshop at the International Congress of Nutrition 2022

The first opportunity for peer review of the recommendations was during a preconference workshop at IUNS-ICN 22 in Tokyo, Japan, on December 6, 2022. Approximately 2 mo before the congress, we publicized the workshop using the authors’ and the FENS social media accounts, eg, via LinkedIn [9] and invited interested congress delegates to register via an online registration form. We also wrote to colleagues directly to both publicize the event and invite them to register online. Approximately 40 participants attended a session that included a presentation introducing the working group activity followed by an opportunity to participate in providing feedback through the widely used, participatory, theory-based World Café method [10]. For discussion, participants rotated to 3 different tables (Manuscript: 1. Abstract, introduction, and other; 2. Methods; 3. Results and Discussion). In the session, the topic was introduced by the table chair, and participants were asked to provide comments through discussion and write their points on a tabletop (Supplementary Figure 1). The chair encouraged them to record their feedback and to comment on each other’s points. Using this approach, the comments from all 3 subsequent groups were discussed in an integrated manner. Finally, all feedback was compiled, and the main points described in Box 1 and the following paragraphs. Full details are available in Supplemental Material. This specific feedback has been used to refine recommendations for a nutrition extension to CONSORT published in our first manuscript [6], and is indicated in italics in Table 1.

Box 1 **Potential key areas in which to improve rigor in nutrition research**

• Using the checklist in study design
• Standardizing registration of nutrition trials and nutrition trial protocols
• Quality rating system for nutrition trials
• Blinding to interventions
• Stratification
• Intention-to-treat vs. per-protocol analysis

Alt-text: Box 1

Participants were very supportive of efforts to improve rigor in nutrition research, and especially, for reporting of trials. They expressed that a nutrition-specific extension to the CONSORT statement is long overdue. They recognized the need for the whole community including scientists and journals to adopt guidelines specific for nutrition trials. They identified additional topics worthy of discussion for improving rigor in nutrition research beyond the edits to the table indicated in Table 1, which are summarized in Box 1.

### Using the checklist in study design

A nutrition (NUT) extension for CONSORT was felt to be long overdue. The proposed checklist along with facilitating adequate reporting in nutrition trials was also thought to be a useful aid at the point of designing studies. Hence participants at the IUNS encouraged the adoption of the proposed checklist by the nutrition community and journal editors, as the move could potentially improve both study design and reporting in nutrition.

### Standardize registration of nutrition trials and nutrition trial protocols

Several discussion points relating to the registration of nutrition trials were shared by attendees at the IUNS preconference. These discussion points could be broadly categorized as those relating to *1*) trial protocols, content, and presentation and *2*) trial registries.

#### Trial protocols

Although the notion of registration of nutrition trials garnered much support, a difference in the rigor across various current registries was noted and the need for harmonizing randomized controlled trial (RCT) registries was expressed. Some aspects of standardization that were discussed included the addition of the date of registration and the use of standardized abbreviations and simple language. The nutrition trial protocol, it was thought, should justify using an RCT to investigate the research question of interest and the population that was included or excluded in the specific trial balancing potential harms and benefits. Participants expressed that the trial protocol should specify if a pilot study was conducted to define study power and include an a priori statistical plan. It was opined that harmonizing aspects of trial registration would improve the rigor and quality of nutrition trials.

#### Trial registries

Participants deliberated on the implications of RCT protocols being published compared with being registered in trial registries. There was some interest in creating a nutrition-trial-specific registry. There was also an acknowledgment of the challenge in labeling an RCT as a “nutrition” trial given that nutrition aspects could either be the exposure or the outcome in an investigation and the outcomes of dietary interventions are not restricted to diet or nutrient intakes. The open nature of trial registries also was a cause of plagiarism concerns for some of the participants.

### Risk of bias (quality) rating system for nutrition trials

The term “study quality” is often used interchangeably with “risk of bias,” but it is important to distinguish between study quality and risk of bias. The term suggests an investigation of the extent to which study authors conducted their research to the highest possible standards. A study may be performed to the highest possible standards yet still have an important risk of bias. For example, often it is not possible to blind participants or study personnel to a dietary intervention group (eg, vegan dietary pattern). Not blinding diet interventions does not necessarily mean they are low quality, but it also does not mean they are free of bias resulting from knowledge of intervention status [11]. Outcome assessor blinding, however, is a source of bias if not implemented, and as such, needs to be addressed [11]. Moreover, reporting a study (quality) in line with reporting guidelines such as the CONSORT statement (for RCTs) [4] or the STROBE-nut statement [12] does not guarantee absence of bias. Nevertheless, reporting of a study in line with reporting guidelines is important to assess risk of bias, which is an important element when considering quality and strength of evidence.

At systematic review level, the established approach to evaluate the credibility of results from primary studies is the risk of bias assessment [13]. The risk of bias of a single RCT or RCTs included in a systematic review should be assessed with a well-established and validated instrument, such as the risk of bias tool by Cochrane [14]. Within the Cochrane risk of bias tool for RCTs, risk of bias is assessed for 6 domains: *1*) selection bias, *2*) performance bias, *3*) attrition bias, *4*) detection bias, *5*) reporting bias, and *6*) other biases (eg, carry-over effects in cross-over trials) [10]. In a previous analysis of 50 (18% of them Cochrane Reviews) randomly selected nutrition-specific systematic reviews of RCTs [15], it was shown that 70% used the Cochrane risk of bias assessment tool [14,15].

Recently, the Cochrane risk of bias 2.0 tool has been published [16], and consists of the following domains: *1*) bias arising from the randomization process; *2*) bias due to deviations from intended interventions (blinding of participants and people delivering the interventions in dietary RCTs is often impossible; but if study participants highly adhere to interventions, RCTs can still be judged as low risk of bias); *3*) bias due to missing outcome data; *4*) bias in measurement of the outcome, and *5*) bias in selection of the reported result. The overall risk of bias for the result will be the least favorable assessment across the domains of bias. Compared with the Cochrane risk of bias tool (study level assessment), published in 2011, the assessment will be conducted at an outcome level.

Dietary adherence has not been included as a specific risk of bias domain in the Cochrane risk of bias tool [14]. However, in the Cochrane risk of bias tool 2.0 [16], nonadherence to a specific dietary intervention can be evaluated within the bias domain “assessing deviations from intended interventions” and varies according to whether review authors are interested in quantifying the effect of assignment to intervention (intention-to-treat effect) compared with effect of adhering to intervention (per-protocol effect) [16]. A recent meta-research study investigated if and how Cochrane nutrition reviews assess dietary adherence to a specific dietary regimen [17]. Several reviews included in the meta-research identified in the meta-analysis added a new risk of bias domain for dietary adherence; however, this approach was not recommended by the authors, because dietary adherence is not a standalone risk of bias domain within the Cochrane risk of bias 2.0 tool [16]. Recently, a new toolkit has been developed for the evaluation of risk of bias specifically in nutrition studies. Nutrition Quality Evaluation Strengthening Tools (NUQUEST) combines nutrition-specific criteria with reliable and validated generic assessment tools to provide a transparent and consistent methodology for the evaluation of risk of bias related to dietary exposure assessment. In its evaluation on 45 studies, NUQUEST ratings had a 93% perfect or near-perfect agreement with published ratings. Where there was disagreement, the nutrition-specific component was a contributing factor in discerning exposure methodological issues [18].

### Blinding

Blinding and allocation concealment were considered 2 of the more challenging design considerations for human nutrition trials. As discussed in our previous work [6], blinding of food, beverage, and dietary supplement interventions with obvious sensory attributes that are difficult to mask and difficult to match with comparators can seem impossible. Nevertheless, efforts to do so have sometimes been woefully inadequate. Product development can provide nearly indistinguishable flavors, colors, odors, and textures that duplicate the intervention. Unintended effects on outcome measures should be carefully considered, such as any texture modifier added to a control might alter the gut microbiome. Participants at the IUNS-ICN 22 preconference also gave suggestions for lessening participant awareness of the intervention through incomplete disclosure. For example, a study could be described as a fruit and vegetable study rather than a study to compare high and low polyphenolic-rich fruits and vegetables.

### Stratification

Stratification in clinical trials is particularly important for smaller RCTs (<400 participants), large trials when interim analyses are planned in subgroups, and those designed to compare the efficacy or effectiveness of several interventions [19]. As diet and nutrition trials typically involve small sample sizes, reporting the stratification approach is important to assess the findings of the trial [19]. For nutrition interventions, stratification based on status or intake of the bioactive/food/dietary pattern or habitual eating behavior is recommended, as responsiveness is likely to be greater in those with less-favorable baseline status. However, to do this, detailed measures of food and nutrient intakes at baseline will be needed. New technologies to support these measures such as ASA24 or myfood24 are now available for researchers’ use. The rationale and methodology for the stratification approach should be well described in the manuscript to aid with the interpretation of the results.

### Intention-to-treat compared with per-protocol analyses

Participants expressed the need to ensure reporting clarity in the data analysis approach, regarding intention-to-treat compared with per-protocol analyses. Intention-to-treat analysis will analyze participants as assigned originally to the study arms, regardless of the treatments received. It is a data analysis approach to assess efficacy as it will respect the randomization process and account for potential confounding. For nutrition trials where attrition can be substantial, the intention-to-treat analysis will need to consider missing observations. When imputations are used to account for missing observations, the imputation approach needs to be reported clearly, including assumptions regarding the nature of the missing values, the type of imputation method used, and number of imputed data sets generated, and so on [20].

A per-protocol analysis may provide complementary information regarding the effect of the interventions. The per-protocol analysis will analyze participants based on the treatment received, regardless of the allocation process. For nutrition trials with considerable dropout and challenges regarding accurate assessment of compliance, per-protocol analysis will provide relevant insights into the effect of the interventions in adhering participants and indicate feasibility of translation of a recommendation to a particular dietary recommendation. As pointed out by Weaver and Miller [21], the more complex the design of menus, and procurement, storage, and transfer of the intervention to the participant, the more problematic for participants to handle receipt of the intervention, including storage, preparation, and protocol compliance. In this respect, compliance is easier to achieve in both dietary supplement and drug intervention trials than in dietary interventions.

The per-protocol analysis can complement the findings of intention-to-treat analysis. The per-protocol analysis can provide evidence regarding the effect of a treatment taken in a specific dose or procedure. The per-protocol analysis, however, may not account for all confounding factors and introduced additional risk of bias. In any case, the per-protocol requires a clear description of the criteria and cutoffs used to assess eligibility, compliance to the treatment, and assessment of outcomes.

Authors of reports on nutrition trials will need to describe and justify the data analysis approach clearly in a statistical analysis plan that is made available a priori to the trial. Any deviations from the plan need to be reported in the research manuscript and poststatistical analysis plan analysis should be identified as exploratory. Finally, a sensitivity analysis approach to interpret findings from an intention-to-treat and per-protocol analyses, is discouraged as both approaches are conceptually different.

## Input from Nutrition Journal Editors

On February 22, 2023, the American Society of Nutrition hosted a Zoom meeting of nutrition journal editors. Eighteen invitations were sent, 6 editors attended, and 2 other editors who could not attend followed up with written input. Editors were provided a background description of the project and 4 questions with the invitation. The main points addressed in response to the questions during the Zoom meeting or subsequently by e-mail are summarized in Box 2. In response to the editors’ suggestion to propose a simple tool to assess compliance with the Nutrition Extension, we developed the survey in Box 3, which consists of a draft list of 19 simplified questions that could be answered via yes/no/not applicable options. The directive of the nutrition editors was to make a checklist as simple as possible rather than have a question for each item in the checklist extension. Further development of this tool is, however, beyond the scope of the present manuscript.

Box 2 **Summary of responses from the Nutrition Journal editors to questions posed by the FENS working group regarding draft recommendations for a nutrition extension to CONSORT**

1. What is your level of interest in having additional guidance for reporting human nutrition trials?
  • The effort to strengthen rigor, reproducibility, and transparency in nutrition publications is important.
  • A nutrition extension is positive for authors and editors.
  • It is crucial to implement the proposed guidance in an approachable manner that overwhelms authors.
  • The recommendations have promise as a teaching tool.
2. How likely would you be to support this initiative? We are probing for interest rather than official commitment at this stage.
  • If FENS develops a useful tool, some editors would adopt it for their journals and others would strongly recommend but may not require it upon submission.
  • A simplified tool such as a survey with dropdown answers to make it easy for authors to use and useful to editors would encourage adoption.
3. List any items in the table of proposed recommendations for a CONSORT nutrition extension that should be deleted or modified.
  • Defining a cut point for adherence needs to be pre-defined in nutrition trials.
  • Both intention-to-treat and per-protocol analyses provide useful information – the first to indicate if people will comply and the second to quantitate effectiveness of the intervention.

Table 1 reflects other suggestions.

4. Are there any other items that should be added to a nutrition extension to CONSORT?
  • Data management and sharing should be included.

Alt-text: Box 2

Box 3 **CONSORT-Nutrition Extension Compliance Assessment for the journal submission process**

1. Title: Has “RCT” or “trial” been identified in the title?

Yes

No

2. Abstract: Have details of the nature and type of intervention or eating behavior and comparator been identified in the abstract?

Yes

No

3. Introduction: Has the biological plausibility been addressed?

Yes

No

4. Introduction: Where relevant, has the context (dietary guidance/population of interest) been addressed?

Yes

No

Not applicable

5. If secondary analysis, is it clearly stated?

Yes

No

Not applicable

6. Methods: Does the trial design align with the scientific question being addressed as stated in the introduction?

Yes

No

7. Methods: Is the dose and duration of the trial justified for primary and secondary outcomes?

Yes

No

Not applicable

8. Methods: Are potential confounders included (relevant baseline nutritional status, participant or environmental effectors, carry-over effects)?

Yes

No

Not applicable

9. Methods: Are nutritional/physiological eligibility criteria included?

Yes

No

Not applicable

10. Methods: Is the intervention fully described (form, preparation, source, matrix, co-ingested components, storage, bioactivity, biological exposure, acceptability and tolerance, how protocol developed for behavioral studies)?

Yes

No

Not applicable

11. Methods: Is the dietary comparator fully described (source, isocaloric, physical/sensory similarity to intervention)?

Yes

No

Not applicable

12. Methods: Was randomization based on nutrient intake?

Yes

No, but justified

Not applicable

13. Methods: Was allocation described?

Yes

No

Not applicable

14. Methods: Was blinding/limits to blinding fully described (nature and who was blinded, ie, participants, staff who delivered the intervention, analytical staff), or if blinding not possible, are steps taken to avoid bias described?

Yes

No

Not applicable

15. Results: Do primary outcomes followed by secondary outcomes follow trial registration and include clinical, public health, and statistical relevance as appropriate?

Yes

No

16. Results: Are ancillary analyses fully described (pre-specified or exploratory, reporting interaction terms, sensitivity analyses, and data imputation where relevant)?

Yes

No

Not applicable

17. Discussion: Has key nutritional relevance been adequately described, ie, generalizability with consideration of background diet and population, differentiating between efficacy and effectiveness, intent-to-treat vs. per-protocol, statistical vs. clinical relevance?

Yes

No

18. Discussion: Have influencing factors been fully discussed (choice of comparator, potential biases, dietary adherence, active constituent, statistical adjustments)?

Yes

No

19. Are data made publicly available?

Yes

No

Alt-text: Box 3

## Grading of Selected Nutrition Trials against Recommendations for CONSORT Nutrition Extension Checklist

Our recommendations for CONSORT nutrition as suggested earlier [6] were used to score the scientific publications in a purposive sample of 8 nutrition RCTs with differing study designs. Through this process, we illustrated the application of our recommendations for improving reporting for primary trials and secondary analyses. Each of these trials was selected by members of the author team, being trials known to them and of differing design. Each trial was independently scored by at least 2 reviewers and discussed and revised in group meetings. Discrepancies arising while scoring using the proposed recommendations were resolved through discussion. Table 2 shows the score for each trial per item on the checklist proposed in Table 1. Scores ranged from −1 to 2, with −1 indicating misinformation, 0 that the information was absent, 1 that the parameter was alluded to but lacked sufficient detail, and 2 indicating that the parameter was adequately covered. The rationales for the scores are given in the text below. Reporting of most trials assessed was strong in some of the parameters in the recommendations presented for a nutrition-specific CONSORT extension, but absent or weak in other parameters considered important in the nutrition extension or from the original CONSORT reporting guideline. The present assessment is by no means definitive but is meant to illustrate the value an extension could provide to better describe and guide the reporting of diet-related interventions. Indeed, these articles all passed peer review in well-regarded journals, and often highly cited and/or significantly advanced the knowledge base in the field, but information gaps specific to nutrition hamper the replication and translation of the research. Deficiencies in the original CONSORT checklist will not be helped by nutrition-specific trial guidance; rather, it is the feedback provided by rigorous reporting that will inform such guidance, as described earlier and in Figure 2.

**TABLE 2:** Evaluations of published diet-related and nutritional trials using the proposed nutrition-additions to the CONSORT checklist^1^

Section	Recommendations for nutrition trials	WHI-Ca/vit D [22]	WHI-fat [24]	DASH-Na [29]	VITAL [30]	PREDIMED [32]	MISAME3 [35]	UPBEAT [36]	NNS [37]
Title and abstract									
Title	The intervention and primary finding are clearly stated and RCT specified	0	1	1	0	1	1	1	1
Abstract	The intervention (composition of the food/supplement/pattern) and how the intervention is delivered (supplements, food, guidance, eating behavior), along with dietary adherence methods	2	1	0	1	2	1	1	2
	Clinical/population relevance is stated.	2	1	2	2	1	2	2	2
Introduction									
Background and objectives	Summary of relevant nutrition-related research Indicate strength and quality of evidence Biological plausibility Population affected	1	0	1	1	1	2	2	2
	If relevant, current population/group recommendations or intake is given	2	0	2	2	0	2	0	0
	Length of intervention justified	0	0	0	0	0	0	0	0
	Purpose of the study and novelty clearly stated	2	1	2	1	2	2	2	2
Methods									
Trial design	The trial design aligns with the scientific question being addressed Duration of the trial appropriate for nutritional outcomes Potential contaminants described	2	0	1	2	2	2	2	1
Participants	Eligibility related to baseline nutrition status Eligibility for settings and locations if applicable	0	0	2	0	1	2	2	1
Interventions	Details of the diet-related intervention Comparators described Compliance assessment clear	2	1	1	1	2	2	2	1
Safety, adverse events	Pertaining to diet-related intervention	2	NA	2	1	1	2	1	2
Outcomes	Biomarker of exposure described	2	NA	2	0	2	2	2	2
Randomization	Randomization based on nutrient intake or status if relevant (eg, with small sample sizes)	0	0	NA	0	0	2	NA	2
Blinding	Blinding issues with diet described	0	0	2	2	2	2	0	2
Results									
Participant flow (a diagram is strongly recommended)	Flow chart included Diet-related dropouts described Reasons for deviations from the protocol	2	1	0	0	1	2	2	2
Baseline data	Consideration of baseline nutrition status	2	0	2	0	1	2	NA	2
Numbers analyzed	Per-protocol/intention-to-treat analyses described	2	0	2	2	2	2	2	−1
Outcomes and estimation	Group differences and variance adequately described	2	2	2	2	2	2	2	−1
Ancillary analyses	Interaction terms, sensitivity analysis, and data imputation described	2	2	2	NA	2	2	2	2
Discussion									
Limitations	Accounted for choice of the comparator, lack of or partial blinding, and quality control, and insufficient power for proposed analyses, including secondary analyses	1	1	1	2	2	2	1	1
Fidelity	Delivery of intervention as intended	2	0	1	2	2	2	1	2
Compliance	Report participant compliance	2	0	2	2	2	2	1	0
Generalizability	Efficacy or effectiveness and generalizability adequately described	2	2	2	2	2	2	2	1
Interpretation	Translation clear	2	1	2	2	2	2	2	−1
Other information									
Protocol	Data accessibility	0	0	2	0	2	2	1	1

DASH-Na, Dietary Approaches to Stop Hypertension-Sodium trialMISAME3, MIcronutriments pour la SAnté de la Mèreet de l’Enfant-3WHI-Ca/vit D, Women’s Health Initiative calcium and vitamin D trialWHI-fat, WHI Dietary Modification Trial: low-fat diet and cardiovascular disease

1 Key: −1 = misinformation, 0 = absent, 1 = alluded to but lacks sufficient detail, 2 = adequately covered.

### Women’s Health Initiative calcium and vitamin D trial

Strengths of the Women’s Health Initiative (WHI) Ca and vitamin D trial report [22] were many, yet reporting fell short of the recommendations in Table 1 for numerous items. The title of the publication did not include the intervention (RCT not stated) or the study population (healthy postmenopausal women) and could have described a review article. This deficiency remains a shortcoming of the *New England Journal of Medicine* and other prominent journals as will be shown below with more recent trials. The introduction did not include biological plausibility for the intervention but did state that the purpose was to test the primary hypothesis that calcium and vitamin D supplementation would lower the risk of hip and other fractures. Nowhere in the article is the number of clinical sites or geographical representation of the participants described. Randomization by nutrient status was not considered, although generalizability was likely achieved with the large trial population. On average, the participants had intakes near recommended levels of calcium and vitamin D status. Enrolling participants with adequate nutrient status relevant to the intervention has been challenged as participants are less likely to respond to the intervention [23]. The active intervention (1000 mg/d calcium as calcium carbonate plus 400 IU/d vitamin D_3_), instructions for consuming the supplements (2 tablets/d in divided doses with meals), and duration of the intervention were adequately described. The rationale for the doses selected, but not the duration, of the intervention (until the study stopped) were given. Adherence to the intervention (weight of returned pill containers) was clearly stated. However, the nature of the placebo comparator was not described nor was blinding discussed.

The statistical analysis plan considered baseline intakes of calcium and vitamin D and vitamin D status by measuring serum 25(OH)D in a subset of the participants, contaminants (especially use of hormone therapy), and intention-to-treat analysis specified in the protocol. Although a flow chart was included, the results included no explanation on whether the 684 participants who withdrew were related to the intervention. Consideration of the errors in self-assessment of dietary intakes, issues concerning blinding and placebo, and lack of eligibility criteria on baseline status of calcium and vitamin D were not discussed when interpreting the findings. The discussion included the possibility that doses of the intervention may have been too low to achieve a benefit. Although calcium and vitamin D resulted in some benefits, the effects were modestly described as possibly related to a lower rate of incidence of hip fracture than predicted, which reduced power to 42%. Data availability was not discussed. Deficiencies partly reflected expectations at the time of the study or journal requirements, but an assessment illustrates the value an extension could serve to better describe diet-related interventions.

### WHI Dietary Modification Trial: low-fat diet and cardiovascular disease

This WHI Dietary Modification Trial [24] describes a secondary analysis from the WHI and raises important questions regarding the level of RCT protocol detail that should be included in such ancillary analysis articles (Table 2). As a minimum, details regarding participant characteristics, group sizes, intervention design and delivery, primary and secondary outcomes, and fidelity and compliance to the intervention, which precludes the need to refer to the primary outcome or trial protocol article, should be included. This article reported on the intervention and post-intervention effects of following a low-fat dietary intervention (target of 20% of energy from fat) on cardiovascular disease (CVD) incidence according to baseline CVD and blood pressure status. Scores were low (often 0 or 1 from a range of −1 to 2) for the proposed additional parameters to CONSORT for nutrition trials. The primary research finding/outcome is not stated in the title. Although the abstract describes participant characteristics well, RCT design and follow-up and key results, information on how the intervention was delivered and dietary adherence assessment methods are absent, with the conclusion only generically describing the clinical or population relevance of the key findings. Of the 4 checklist recommendations regarding the introduction content for diet-related RCTs, only the purpose of the study and novelty are alluded to. However, the originality in approach and rationale for this further analysis relative to the previous relevant WHI outputs [[24], [25], [26], [27], [28]] is not clearly described. Apart from these previous WHI study publications, no summary of the relevant literature on the effect of low-fat diets on CVD outcomes was included. Although some detail on dietary targets and the delivery of the intervention is included in the methods, key information such as the length of the intervention and eligibility related to baseline nutrition status are absent. The text describing dietary intake and adherence is vague: “Dietary intake was monitored by obtaining periodic food frequency questionnaires (FFQs) and by performing laboratory analysis of blood specimens for a subsample of women (5.8%),” with no information given as to the timing of the assessments, the key criteria considered in the FFQ, or the biomarkers quantified in the blood samples, and thus the reader must refer to other sources to obtain this information. The brief statistical section does not sufficiently describe what constituted per-protocol analysis. The results are clearly and comprehensively described. However, no information is given if the dropouts were diet-related, if there were deviations from the protocol, whether the results of per-protocol compared with intention-to-treat analyses agreed. Furthermore, the response to intervention was not stratified according to the baseline nutrition status or adherence to the intervention. Although, the discussion considers mediation analysis to examine the influence of postrandomization differences in dietary variables on coronary heart disease hazard ratios that contrast the intervention group with the comparison group, unusually these data are not presented in the results section. In this secondary analysis article, there is no discussion of the fidelity of the intervention or participant adherence.

### Dietary Approaches to Stop Hypertension-Sodium trial

The Dietary Approaches to Stop Hypertension (DASH)-Sodium trial [29], despite being published before the inception of CONSORT, is a comprehensive and informative report. Nevertheless, the article could have benefited from increased detail as defined by the recommendations for a nutrition-specific CONSORT extension discussed herein. The strengths of the DASH report lay in a clear purpose statement for the research and corresponding completeness of results, missing only the CONSORT flow chart and additional detail on dropouts and deviations, likely due to the age of the article and journal requirements at that time. The title of the article did not indicate the study design or primary finding, and the introduction could have been strengthened by a better summary of supporting research, including a justification for the intervention period. The methods section was adequate, including clear descriptions of population groups included, the eligibility criteria, safety of the intervention of assessment of safety measures, definition of the biomarker of exposure (urinary sodium), and blinding. Details on obtained ethics committee approval were given. The methods, however, lacked sufficient detail on trial duration, details of the comparator diet and compliance measurements and thresholds used. Instead, the reader was directed to previously published research. The results section lacks information about the recruitment setting, and the overall findings reported only the results from intent-to-treat analysis, which was based on using imputing missing data with baseline values. Reporting adherence measures would have facilitated a better interpretation of the results. The discussion, although articulate, lacked details on the choice of comparator, blinding, and quality control, as well as a discussion on intervention fidelity. Potential mechanisms of action of the intervention effects were not discussed. Overall, the interpretation of the results and their generalizability were appropriately presented. There was no clear declaration of conflicts of interest aside from listing study sponsors.

### Vitamin D and Omega-3 Trial

The VITamin D and Omega-3 Trial (VITAL) [30] investigated the impact of long-term supplementation on cancer and CVD-related outcomes. The VITAL study was presented in 2 articles, in which the impact of vitamin D and omega-3 supplementation were presented separately [30,31]. Per our criteria, VITAL was rated as moderate, as several essential elements inherent to a strong dietary intervention were either weak or absent (Table 2). Although this was a robust study conducted in a large US cohort, the main outcome/primary research finding is not cited in either article title. Although the clinical relevance of the population was clearly stated in the abstract, key details in relation to the supplements and any biomarkers of compliance were lacking. In the introduction, the population relevance was well articulated, however, key background details pertaining to the current state of the art of the relevant nutrition-related research and biological plausibility were lacking. A clear and strong RCT design is strength of VITAL, and Figure 1 in the vitamin D article presents essential elements related to screening, randomization, and follow-up of the study cohort. Nevertheless, baseline vitamin D and/or long chain n–3 polyunsaturated fatty acid (LC n–3 PUFA) status, which would no doubt affect the outcome, were not captured in the methodology. Presumably, this study was conducted in several centers, however, details were not provided. In terms of monitoring the intervention, VITAL lacked robust biomarkers of compliance, which would have accurately assessed actual intervention/exposure to the nutritional intervention. VITAL reported adverse events that included hypercalcemia, kidney stones, and gastrointestinal symptoms in the vitamin D article and gastrointestinal symptoms and major bleeding events in the omega-3 article. VITAL took an intention-to-treat statistical approach, and all outcomes of the statistical analysis are clearly described. The discussion presents the findings in a balanced fashion within the context of previous studies, including the presentation of negative findings. Also, the authors do acknowledge potential limitations in terms of a single dosage of vitamin D and LC n–3 PUFA, as well as a relatively short intervention time of 5.3 y follow-up, given the pathological progression from health to incident disease can often extend over decades for both disease outcomes.

#### Prevención con Dieta Mediterranea trial

The Prevención con Dieta Mediterranea (PREDIMED) study [32] was a multicenter RCT comparing a Mediterranean Dietary Pattern enriched with either extra virgin olive oil or mixed nuts with a low-fat diet control on cardiovascular events in at-risk individuals. A detailed description of the methodology was presented initially as a cohort profile: design and methods of the PREDIMED Study [33] with the primary outcome article [34] containing a supplementary appendix with additional protocol information. These articles are accompanied by a research protocol available on the PREDIMED study website (www.predimed.es). The accessibility of this information is a major strength of this trial. A trial of this magnitude together with intervention resources are valuable tools for readers. The article published based on a re-analysis, Estruch et al. [32], has been used to review PREDIMED for this opinion piece. PREDIMED was a relatively robust trial report with more than 65% of elements inherent to a strong dietary intervention (score of 2) adequately covered (Table 2). The article title did not indicate that it was an RCT or clearly state the main finding. The relevance of the trial for a clinical population was alluded to but additional detail around impact and significance could have been added. The abstract provided excellent detail about the dietary intervention. The introduction provided an overview of the rationale for the intervention, previous research, and the potential benefit of the findings from a public health perspective. Biological plausibility was not clearly articulated. Given the population is Spanish, it may have been more appropriate to include the baseline adherence score (average 8–9 out of a 14-point Mediterranean dietary adherence score) in the main article rather than as supplementary information. Although there was no justification for the length of the intervention, the authors described the statistical power required and length of trial and subsequent use of stopping boundaries and *P* values for stopping the study at each yearly interim analysis for adverse effects. There was no eligibility related to baseline nutrition status stated in the article, rather eligibility was based on age and other cardiovascular risk factors. Settings and locations of the trial centers were described, but the eligibility of clinics that participated was not explained. Presumably, these clinics were selected based on the characteristics of patients that attended that matched the intervention eligibility criteria. A detailed flow chart was included in supplementary material; however, the reasons for withdrawals were absent. Although a dietary intervention trial that manipulates whole diets cannot be blinded to the participant or investigator, the PREDIMED report described the blinding of various outcome measures and committees. The end point adjudication committee that examined medical records related to end points was blinded to the intervention group randomization. A particular strength of this study is the detail of the dietary intervention and comparator intervention together with measures of dietary adherence. Adverse events relating to dietary intervention were captured in yearly questionnaires. Similarly, dietary issues with the intervention such as difficulty with chewing nuts were dealt with by offering alternative consumption methods. Statistical methods were well outlined; the analysis was conducted based on the intention-to-treat with per-protocol cutoffs adequately described.

#### MIcronutriments pour la SAnté de la Mère et de l’Enfant-3

De Kok et al. [35] presented the findings of a randomized controlled efficacy trial [MIcronutriments pour la SAnté de la Mère et de l’Enfant (MISAME)-3] that compared the effect of a fortified balanced energy protein supplement against a standard of care iron and folic acid supplement in a population of pregnant women in Burkina Faso. The effects on birth outcomes (as one of the primary outcomes) were reported in the present manuscript, together with other secondary outcomes at birth. The second primary outcome (child growth at 6 mo) will be reported in a separate manuscript, together with the findings of the intervention during the lactation period. The findings of the biochemical analysis will also be published separately. Although a combination of all outcomes in one manuscript would have been informative, it could have led to a lengthy article and probably delayed the publication of the findings considerably. Describing the approach to publish the different findings, however, was informative and will help orientate the reader toward other findings of the trials.

The trial was reported with sufficient detail, although the description of the specific primary outcome and composition of the supplement in the intervention group could be improved in the title and abstract, respectively. The length of the intervention was not specified. The timing of introducing the supplement during gestation, however, was reported. Overall, the remainder of the manuscript contained sufficient detail on conflicts of interest, availability of a study protocol, ethical approvals and an a priori statistical analysis plan, with details of per-protocol analysis, cutoffs, procedure to assess adherence, and ancillary analyses. Although the effectiveness of the intervention was described in detail, the article did not contain an explicit statement on the generalizability of findings.

Strength of the article is the availability of supplementary materials that contain the findings of complete case, per-protocol, and subgroup analyses by potential treatment modifier. A specific website is provided where most study material such as questionnaires and details of the procedures followed can be downloaded freely. The participant-level study data could not be shared due to the informed consent procedures. In the absence of a data access committee, a contact of the ethics committee is provided to handle data requests.

### UK Pregnancies Better Eating and Activity Trial

The UK Pregnancies Better Eating and Activity Trial (UPBEAT) is an RCT comparing a diet and physical activity behavioral intervention with standard antenatal care on the incidence of gestational diabetes and large for gestational-age infants [36]. The UPBEAT study scored mostly 2 and 1 (Table 2). The delivery of the intervention is well described in the abstract section; however, there is no description relating to the composition of the diet and physical activity behavioral intervention. Only at the end of the abstract did it become clear that diet and physical activity were the key components of the intervention. Similarly, the primary outcome of the study was not presented in the title but appears at the end of the background section. Of the 4 recommendations proposed for the CONSORT nutrition extension (Table 1), UPBEAT did not report on current population recommendations or dietary intake or justify the length of the intervention within the antenatal period. There was a detailed discussion on research, mainly from systematic reviews and quality of evidence. The length of intervention was not justified, and there is little explanation on biological plausibility. What is missing is information on usual or current care for this group. NICE guidelines are mentioned in the methods section, in terms of early screening for glucose intolerance but not explained. The reader is referred to a previously published protocol for recruitment details. Several factors noted in the Nutrition Extension CONSORT may not be completely relevant for behavioral interventions. In the UPBEAT study, the outcomes are described extremely well, but there is no biomarker of exposure. The primary outcomes were the presence of gestational diabetes and the incidence of large-for-gestational-age infants. Similarly, randomization was done by minimization of nonnutritional factors. Blinding of participants and investigators to a behavioral intervention is impossible; however, there was no mention of blinding relating to analyses of outcomes. Furthermore, baseline nutrition status was not particularly relevant in this study as BMI was the key consideration. For the 5 topics listed in the consensus recommendations for discussion, reporting of UPBEAT had little information on limitations, fidelity, and compliance to the intervention, key reporting criteria for behavioral interventions.

### Nonnutritive sweetener “omics” trial

The nonnutritive sweetener (NNS) RCT on glucose tolerance and changes in the oral and fecal microbiome and plasma metabolome represents a study using “omic” tools to explain individual responses to a diet intervention [37]. The precision nutrition paradigm is based on the premise that substantial variation exists between subjects in terms of diet-related disease risk and response to dietary interventions. In terms of advancing the state of the art, it is essential that the magnitude of interindividual variability in response to any nutrition supplementation or intervention, after accounting for sources of variability not attributable to supplementation, is characterized. Within-subject variability includes variability attributable to changes in the outcome due to chronic biological, behavioral, or environmental changes that are unrelated to the intervention, including any number of coincidental lifestyle factors. Importantly, within-subject random variation can be so large it may explain most, if not all, the apparent interindividual variability in response to the intervention. The NNS trial illustrates a great need to standardize reporting and develop good practices for data sharing. So much data are generated with “omics” approaches that many discoveries could occur for many years if made publicly available.

The title lacked a description that it was an RCT. Background diet information of the participants was not reported, but eligibility criteria included non-NNS use. The source of the NNS for the trial was not given. There was no apparent statistical analysis plan, and the main outcome measure (glucose tolerance test) was not the main outcome listed in the clinicaltrials.gov, ie, continuous glucose monitoring, which may be more related to the gut microbiome than an acute tolerance test. Dietary contamination was considered and key covariates such as age, gender, BMI, and smoking were described. Responders were compared with nonresponders, but there was no a priori definition of criteria for determining a responder and sometimes 3 were chosen and sometimes 4, which appeared to be cherry-picking and risked breaking randomization. For a characteristic of “responder” or not, a repeated test would be needed to determine if the response was within normal variation, particularly as there is overlap between groups. This would be specifically important for an outcome such as the glycemic response, which would have a high within-person coefficient of variation. Some perspective of clinical relevance of a statistically significant effect on glucose area under the curve is warranted. Thus, lacking clear information, a score of −1 was given for numbers analyzed, group difference determinations, and translation of results. Two authors disclosed key relationships with personalized nutrition companies that may benefit from the publication of this manuscript, and 1 author was a member of the scientific advisory board of the journal in which this trial is published. In summary, the reporting of this trial scored poorly on many of the features we propose for consideration in a CONSORT checklist focused on nutrition trials (Table 2), but personalized nutrition is a timely area, and this study is seen as advancing the field. It is imperative to formulate reporting guidelines as the field progresses forward using this exciting approach.

## Conclusions and Inviting Further Input

The perspectives offered herein provide some evidence for the desirability, timeliness of a nutrition extension to the existing CONSORT guidelines, and the general acceptability of such guidelines by the nutrition community and respective journal editors. We recognize that many of the deficiencies we identified are not unique to nutrition trials, but a recent review of nutrition trials illustrates the extent of compliance with current CONSORT guidelines [38]. Of 400 trials reviewed, only 69% were registered and many of these lacked sufficient details of outcomes and treatment effects and information to inform risk of bias assessment, Protocols were available in only 14% of the trials and statistical analysis plans in 3%. We investigated the applicability of a nutrition extension to CONSORT to improve the reporting quality of trials conducted in the nutrition domain that have previously relied on CONSORT for their reporting. Through both peer-led discussions as well as those with the editors’ group, we are cognizant of the need to keep the burden on authors, editors, and reviewers low, such that the final guidelines, although comprehensive, should be kept as short as possible.

The next steps are to initiate a formal guideline development process with stakeholders, per guidelines development recommendations [8]. Our proposal may need further refinement in line with updates to the CONSORT guidelines that are currently ongoing [39], yet through this present article and our previous manuscript, we hope to have triggered interest and engagement in an initiative to improve the reporting of RCTs in nutrition.

### Acknowledgments

We thank Philip Calder, Jan de Vries, Isabelle Herter, Tracy McCaffrey, and Katherine Tucker for their help in running the World Café workshop at the IUNS Congress, December 2022, as well as all Congress delegates who gave their time and inputs in this session. We additionally wish to thank the journal editors who responded to our invitation and provided inputs in the call and via e-mail. We would also like to acknowledge Tesfamariam Hadush and Yves Didier Umwungerimwiza for their assistance in the assessment of the reporting completeness of the MISAME article.

### Conflicts of interest

SA receives a salary from BioActor BV. JC is Director of Dietary Assessment Ltd. SS has received consulting fees from Abbott Laboratories Sdn Bhd. CW is involved in training of best practices in the design, conduct, and documentation of human nutrition trials for the *American Society for Nutrition*. JC and CL were involved in developing the STROBE-nut statement for reporting observational studies in epidemiology focusing on nutrition. CL is a co-author of the MISAME 3 study that was assessed as an example of reporting completeness and is the PhD supervisor of Kokeb Tesfamariam Hadush and Yves Didier Umwungerimwiza who contributed to the assessment of the MISAME study for the present manuscript. LS is a member of the GRADE working group. JR-F has published and, at the time of writing, currently works on secondary analyses of samples and data from the UPBEAT study that was assessed as an example of reporting completeness. All other authors report no conflicts of interest.

### Author contributions

The authors’ responsibilities were as follows **–** CW, JR-F: designed the review with considerable inputs from CL, A-MM, KJM, SS, and SA; CW: had primary responsibility for final content; and all authors: contributed to drafting the manuscript and read and approved the final manuscript.

### Funding

The authors reported no funding received for this study.

## Figures and Tables

**Figure:**
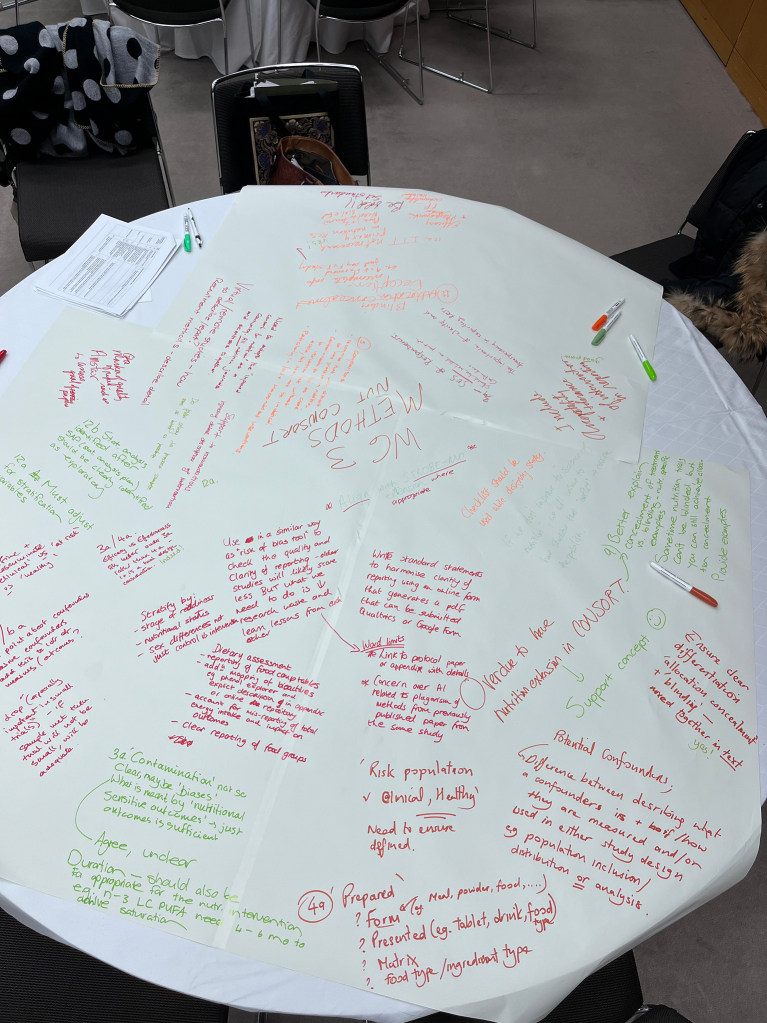
Supplementary Figure 1: Example "World Café" method brainstorming tablecloth resulting from discussions on a CONSORT extension for nutrition trials at the International Union of Nutritional Sciences International Congress of Nutrition, held in Tokyo, Japan, Devember 6, 2022.

## Appendix A Supplementary data

Multimedia component1 Multimedia component1
