# Supplementary material for: Perspective: Peer Evaluation of Recommendations for CONSORT Guidelines for Randomized Controlled Trials in Nutrition
Source: Adv Nutr. 2023 Nov 21;15(1):100154. doi: 10.1016/j.advnut.2023.100154 (PMC10716705; doi:10.1016/j.advnut.2023.100154)
Supplement: Multimedia component1 [file mmc1.docx]

**Perspective: peer evaluation of recommendations for CONSORT guidelines for randomized controlled trials in nutrition**

**Connie Weaver**

**Findings from participants of the Symposium at ICN-IUNS 2022**

**7 December 22, Tokyo**

Note that the following paragraphs present findings from participants who attended the peer feedback symposium at the International Union of Nutritional Sciences International Congress of Nutrition on 7th December 2022. It includes general discussion points on the merit of a CONSORT-nutrition extension, as well as points regarding both general aspects of reporting per manuscript section and on specific items of the consensus recommendations for a nutrition-specific extension to the 25-item CONSORT checklist, previously published by our group (Rigutto-Farebrother et al., 2023).

**General comments on a CONSORT-nutrition extension:**

- The proposed checklist should be used when designing studies
- Need to adopt guidelines as a community-journals and scientists alike
- There was much support for a NUT extension to CONSORT-long overdue
- If we don’t improve reporting requirements, will continue to have weak studies published
- Registration of RCTs? Publishing of protocol? Can we harmonize RCTs registries? Does CONSORT have a role to play, to ensure rigor and clarity? Global registry - there are different systems; with some more rigorous than others.
- Nutrition trials may not be registered within a trial registry; CONSORT could help to standardize this. An example system is provided by clinicaltrials.gov (USA). Could this registry be influenced to include a nutrition study branch? If the primary trial outcome or predictor is a nutrition variable, then registration is a “nutrition” trial. ASN and FENS could petition for use of CONSORT+ as new part of website. (From point 17a. See also #23 on this point). (Some participants noted that it is sometimes difficult to classify only as a nutrition trial as sometimes diet/nutrients are the independent variable and others the dependent variable; with other major aspects of intervention/outcome).
- Align recommendations/definitions with STROBE-nut where possible

**Comments on manuscript elements:**

1. **Title / abstract / introduction**

**Overall comments:**

- Standardization of abbreviations
- “Typology of comparisons” would help, should state if pilot study has been conducted and define power; and should use simple language
- As much as possible should define baseline conditions; if participants are not deficient, what is the potential for response? And therefore likelihood of success (this has plagued many trials and can result in the false impression that the intervention does not work, when secondary analyses show that it does work on those deficient; However, many trials do not measure baseline status, leaving conclusions unclear).
- By their nature trials maximize internal validity and limit external generalizability; should be noted up front.
- Consider ethics; who benefit, who is excluded?
- Someone asked, why an RCT? Design should be justified.

**Comments specific to consensus recommendations (Table 1, main paper):**

- 1a:
  - “Where possible” is important to keep
  - Indicating the comparison is important – agree with suggestion but title could become cumbersome, and journals may have their own rules too.
  - Will journals be willing to adopt long titles?
  - It is important for policy making to be able to judge the level of evidence provided from the cited reference, e.g., mouse studies vs human trials
- 1b:
  - Abstract length is often limited.
  - Can journals be more flexible with word count to 250-280 words?
  - Should we increase word count for abstracts in a CONSORT-nut extension? If so, why is nutrition “special” in this respect?
  - Include details of comparator and group size/portions in first bullet point
  - Group size and participants need to be included
  - Abstract to include treatment effects, not only P values
  - Some types of trials are more difficult to capture in CONSORT terminology, e.g., meal-based experiments, absorption studies, dose response studies, etc.
- 2a:
  - PICO Criteria can be extended to methods section e.g., specialties of the intervention, introduction should be more geared to rationale. PICO criteria are likely touched on when study being rationalized.
  - contextualization “where relevant” (second bullet) – the “where relevant” should be added
  - Which dietary recommendation to refer to? There is not always a dietary recommendation for each study. What if there are no recommendations, e.g., for polyphenols?
  - Plausibility and mechanisms – mostly this is already done.
  - Duration and dose (2b) could be included in first bullet of 2a
- 2b:
  - Should be objectives, aims AND hypotheses. The aim of the study is always to determine something, the objective to test the hypothesis – how you did it.
  - Objectives is better in methods
  - Length discussion should be in 2a and methods and discussion

**METHODS**

**Overall:**

- Develop an online statement submission with drop down categories/standard statements beyond a checkbox for addressing an item but not saying how it was done
- Need to adopt guidelines as a community-journals and scientists alike
- Need to develop a quality rating system for papers similar to the Risk of Bias system for systematic reviews or Amstar to improve quality and clarity of reporting and to learn from each other
- Concern over increased difficulty in publishing about plagiarism with trial design papers for methods-only so many ways to describe

**Specific to table:**

- 3a:
  - Consider stratifying by nutritional status, sex, stage of readiness (for behavioral interventions) -not just intervention and control
  - Effectiveness vs efficacy fits better under 3a than 4a
  - Measure confounders not just list them
  - ‘nutritionally sensitive outcomes’ is unclear; ‘outcomes’ is sufficient
  - Duration should be adequate for nutrition intervention-ex. n3 LC needs 4-6 weeks to achieve saturation
  - Consider social-economic status - important for context and transparency
- 4: Virtual/remote studies - how to report? Describe recruitment in detail.
- 4a
  - What does ‘prepared’ include? Need to consider form, matrix, co-ingested nutrients and constituents, food type, presentation (tablet, drink, food)
  - Expand to clinical, at risk, and healthy populations
- 5:
  - Recognize that comparators should be as similar to intervention as possible but difficult to achieve, especially for foods and macronutrients
  - Align recommendations/definitions with STROBE where can (also applies to other points)
  - Include acceptability and tolerance of intervention
- 6a: Measure anticipated confounders
- 8a: Drop the qualifier of especially in small trials as should obey power calculations to be adequate
- 9: Clearly distinguish concealment of allocation and blinding-re-thing location on table
- 11: Blinding-one strategy is incomplete disclosure to participants ex. This is a fruit and vegetable study rather than a high vs low polyphenolic content F & V study
- 12a:
  - Must adjust for stratification variables
  - ITT is not necessarily highest priority in nutrition studies where determining efficacy and mechanisms and drop out rates high so randomization destroyed. Want to establish effect under per protocol conditions before moving to effectiveness studies. Be bold in leading the way on this recommendation
- 12b: Post SAP analysis should be clearly identified as exploratory

**RESULTS**

- 13a:
  - If intervention administration differs from protocol/design, then outline in supplemental info. Ensure change in protocol also captured in trial register.
  - Drop out in nutrition often also means that you cannot assess the primary outcome – how to do intention to treat analysis? Particularly if binary analysis e.g., got disease/did not get disease
  - Diet interventions tend to have high noncompliance rates; differential dropout; should plan in advance on how to analyze in these conditions
  - Provide power analyses for proposed stratified analyses
- 15:
  - What baseline variables should be measured? BMI, diabetes status, dietary pattern, supplements used, biomarkers?
  - Appropriately, capture dietary lifestyle and use of inclusion / exclusion criteria to include supplement use, diet habits. Nutrition research requires understanding of how behavior, not just physiology, can affect outcomes. Include baseline diet.
  - Baseline data nutritional differences – related to research question e.g. take supplements
  - Record body weight at start and end and report; is unanticipated weight gain an adverse event?
  - Recognize that food interventions involve substitution, and thereby differ from medication trials
- 17a:
  - What is clinically significant? Clinically relevant vs public health relevance vs statistical significance
  - What significance is realistic for some aspects of nutrition that may be contextual?
  - Sensitivity analysis is essential. Analyze all scenarios and identify the analysis a priori in statistical analysis plan. Blinded analysis e.g., with statistician or lab staff.
  - How do we know what to stratify by? Nutritional status or behavioral….
  - Participants? Characteristics by sex
- 19: Include adverse events in publication. Include harms associated with social/psychologic issues.

**DISCUSSION**

- With increasing complexity of analysis approaches, how do trials fit into precision nutrition and systems analysis, AI, etc.

**OTHER**

**Specific to table:**

- 23:
  - Add date of registration.
  - Should there be a nutrition registry? Or amendment to existing one?
  - What about a method bank?
  - Registration should include *a priori* definition of statistics
- 24: Attach protocol, attach questionnaires
- 26:
  - If adhered to CONSORT, then this should be less important
  - Questionable legality
  - We all have certain biases, but the new item may be too intrusive. Goes too far

**KEYWORDS**

**Specific to table:**

- Should be in the list
- Keywords based on PICO?

**References:**

Rigutto-Farebrother J, Ahles S, Cade J, Murphy KJ, Plat J, Schwingshackl L, Roche HM, Shyam S, Lachat C, Minihane AM, Weaver C. Perspectives on the application of CONSORT guidelines to randomised controlled trials in nutrition. Eur J Nutr. 2023 Aug;62(5):2319-2332. doi: 10.1007/s00394-023-03137-5.
